# OXA-181-Like Carbapenemases in Klebsiella pneumoniae ST14, ST15, ST23, ST48, and ST231 from Septicemic Neonates: Coexistence with NDM-5, Resistome, Transmissibility, and Genome Diversity

**DOI:** 10.1128/mSphere.01156-20

**Published:** 2021-01-13

**Authors:** Sharmi Naha, Kirsty Sands, Subhankar Mukherjee, Bijan Saha, Shanta Dutta, Sulagna Basu

**Affiliations:** a Division of Bacteriology, ICMR-National Institute of Cholera and Enteric Diseases, Kolkata, West Bengal, India; b Department of Medical Microbiology and Infectious Disease, Division of Infection and Immunity, School of Medicine, Cardiff University, Cardiff, United Kingdom; c Department of Neonatology, Institute of Post-Graduate Medical Education & Research and SSKM Hospital, Kolkata, West Bengal, India; Antimicrobial Development Specialists, LLC

**Keywords:** OXA-181/232, NDM-5, neonates, sepsis, dual carbapenemases, ColKP3, WGS, core genome, India

## Abstract

Studies on the epidemiology and genomes of isolates harboring OXA-48-like genes in septicemic neonates are rare. Here, isolates producing these carbapenemases which emerged and persisted in an Indian neonatal unit were characterized in terms of their resistome, transmissibility, and genome diversity. Antibiotic susceptibility and whole-genome sequencing were carried out. The sequence types, resistome, virulome, mobile genetic elements, and transmissibility of carbapenem-resistant plasmids were evaluated. Core genome analysis of isolates was shown in a global context with other OXA-48-like carbapenemase-harboring genomes, including those from neonatal studies. Eleven OXA-48-like carbapenemase-producing Klebsiella pneumoniae (*bla*_OXA-181_, *n* = 7 and *bla*_OXA-232_, *n* = 4) isolates belonging to diverse sequence types (ST14, ST15, ST23, ST48, and ST231) were identified. *bla*_OXA-181/OXA-232_ and *bla*_NDM-5_ were found in a high-risk clone, ST14 (*n* = 4). *bla*_OXA-181/OXA-232_ were in small, nonconjugative ColKP3 plasmids located on truncated Tn*2013*, whereas *bla*_NDM-5_ was in self-transmissible, conjugative IncFII plasmids, within truncated Tn*125*. Conjugal transfer of *bla*_OXA-181/OXA-232_ was observed in the presence of *bla*_NDM-5_. The study strains were diverse among themselves and showed various levels of relatedness with non-neonatal strains from different parts of the world and similarity with neonatal strains from Tanzania and Ghana when compared with a representative collection of carbapenemase-positive K. pneumoniae strains. We found that *bla*_OXA-181/OXA-232_-harboring isolates from a single neonatal unit had remarkably diverse genomes, ruling out clonal spread and emphasizing the extent of plasmid spreading across different STs. This study is probably the first to report the coexistence of *bla*_OXA-181/232_ and *bla*_NDM-5_ in neonatal isolates.

**IMPORTANCE** Neonatal sepsis is a leading cause of neonatal mortality in low- and middle-income countries (LMICs). Treatment of sepsis in this vulnerable population is dependent on antimicrobials, and resistance to these life-saving antimicrobials is worrisome. Carbapenemases, enzymes produced by bacteria, can make these antimicrobials useless. Our study describes how OXA-48-like carbapenemases in neonatal septicemic Klebsiella pneumoniae shows remarkable diversity in the genomes of the strains and relatedness with strains from other parts of world and also to some neonatal outbreak strains. It is also the first to describe such resistance due to coproduction of dual carbapenemases, (OXA)-48 and New Delhi metallo-β-lactamase-5, in Klebsiella pneumoniae from neonatal settings. Carbapenemase genes situated on plasmids within high-risk international clones, as seen here, increase the ease and transfer of resistant genetic material. With the WHO treatment protocols not adequately poised to handle such infections, prompt attention to neonatal health care is required.

## Abstract (precis)

Neonatal sepsis is a leading cause of neonatal mortality in low- and middle-income countries (LMICs). Treatment of sepsis in this vulnerable population is dependent on antimicrobials, and resistance to these life-saving antimicrobials is worrisome.

## INTRODUCTION

Neonatal sepsis is one of the primary causes of neonatal deaths (23%) in middle- and low-middle-income countries (1). Multidrug-resistant bacteria complicate the treatment of sepsis in this vulnerable population (2). Klebsiella pneumoniae, belonging to the *Enterobacteriaceae* family, is one such species that has high rate of acquisition of resistance compared to other bacteria of this family (3). In addition, K. pneumoniae is also the leading cause of neonatal sepsis in developing countries (4). With escalating resistance to all available β-lactam antibiotics for neonates (penicillins, monobactam, cephalosporins, etc.), use of carbapenems has gradually increased, ultimately leading to a global upsurge of carbapenem-resistant K. pneumoniae (CR-*Kp*) in the last 2 decades (1, 3). According to the Centre for Disease Dynamics, Economics & Policy (CDDEP), there has been an increase in CR-*Kp* from 24% (2008) to 59% (2017) in India (1), a country that bears the burden of one-fourth of all neonatal deaths that occur globally each year (5).

K. pneumoniae is known to produce different carbapenemases, including Ambler class A carbapenemases (e.g., KPC), Ambler class B metallo-β-lactamases (e.g., NDM, IMP, VIM, etc.) and Ambler class D carbapenemases (e.g., OXA-48) (3, 6, 7). The New Delhi metallo-β-lactamase (NDM) is the most prevalent and worrisome, as it confers resistance not only to carbapenems but to almost all hydrolyzable β-lactams and has rapidly spread worldwide (8). To date, 29 variants of NDM have been reported (https://www.ncbi.nlm.nih.gov/pathogens/isolates#/refgene/ndm). NDM-1 is the most disseminated variant, followed by NDM-5, which was first detected in Escherichia coli from the United Kingdom (9, 10). *bla*_NDM-5_ differs from NDM-1 at two amino acid positions, V88L and M154L, and exhibits enhanced resistance to carbapenems and extended-spectrum cephalosporins (9, 10).

Though NDM has gained prominence, oxacillinase (OXA)-48-like carbapenemases (OXA-48), first reported from Turkey in K. pneumoniae (2001) (6), has now spread to different genera of *Enterobacteriaceae*. Outbreaks and case reports throughout Europe, North Africa, the Middle East, and South Asian countries are increasingly documented (11–13). Reports of emergence or outbreak in neonatal units from Middle Eastern countries have also surfaced (14). Detection of OXA-48-producing microorganisms is not limited to clinical settings and is often detected in environmental surface samples, companion animals, livestock, production animals, and wild animals (11, 15, 16).

To date, 39 variants of OXA-48 have been reported (https://www.ncbi.nlm.nih.gov/pathogens/isolates#/refgene/oxa-48). Currently, OXA-181 and OXA-232 constitutes the 2nd and 3rd most common global OXA-48-like derivatives after OXA-48 (14). OXA-181 was first reported from India (17) and differed from OXA-48 by four amino acid substitutions (T104A, N110D, E168Q, S171A) but did not evolve from it. On the other hand, OXA-232 first reported from France is a derivative of OXA-181 with a single amino acid substitution at R214S (14). OXA-48-like enzyme hydrolyzes penicillins and narrow-spectrum cephalosporins efficiently but does not hydrolyze extended-spectrum cephalosporins and exhibits poor activity toward meropenem while also showing the highest known catalytic efficiency for imipenem (6). Therefore, OXA-48 producers often remain undetected during surveillance because they are categorized as susceptible to carbapenems according to CLSI and EUCAST (6, 14). Like other carbapenemases, OXA-48-like carbapenemases are not inhibited by conventional β-lactamase inhibitors, but nowadays, use of avibactam (a non-β-lactam β-lactamase inhibitor) has been put forward. However, increasing reports of resistance toward avibactam have been documented (18). Hence, specific phenotypic detection of class D carbapenemases is still confusing. High-level resistance to temocillin (MIC, >64 mg/liter) has been suggested as a criterion to screen OXA-48-like carbapenemase; however, due to a similar resistance profile toward KPC and other metallo-β-lactamases (19), this is not suitable. This further emphasizes the difficulty in the identification of OXA-48, which inevitably leads to poor tracking of emergence and spread, and infection control measures. Carriage of such resistance markers on plasmids is often associated with international clones such as sequence type 11 (ST11), ST14, ST15, ST63, ST147, ST231, etc., which aided in their rapid dissemination across boundaries (10, 14, 20).

Studies focusing on the epidemiology and genomic characterization of isolates harboring OXA-48-like genes particularly in neonatal septicemic cases are rare, with few reports of outbreaks or sporadic infections (13, 14). This study, however, monitors the presence of these genes in a neonatal unit over a period of 4 years (2013 to 2016) and evaluates the isolates in terms of their STs, production of multiple carbapenemases, their transmissibility, and associated mobile genetic elements. We performed core genome analysis incorporating isolates in this study in a global context with other OXA-48-like carbapenemase-harboring genomes, including those from other neonatal studies, to explore the genomic epidemiology and variability of carbapenemase lineages, focusing on the context of neonatal sepsis.

## RESULTS

### Bacterial isolates, their susceptibility, and genotypic profiles.

During 2013 to 2016, 195 nonduplicate *Enterobacteriaceae*, including Escherichia coli (*n* = 35, 18%), Klebsiella pneumoniae (*n* = 146, 75%), Enterobacter aerogenes (*n* = 3, 1.5%), Enterobacter cloacae complex (*n* = 11, 5.6%) were identified which were resistant to piperacillin (89%), cefotaxime (80%), aztreonam (78%), and ciprofloxacin (70%). Resistance to meropenem was 47%, whereas few were resistant to tigecycline (2%) or colistin (5%).

Out of 195 strains identified, 11 strains (6%) were found to harbor *bla*_OXA-48-like_ genes by conventional PCRs. Other carbapenemases detected were *bla*_NDM_ (*n* = 73, 38%) and *bla*_KPC_ (*n* = 4, 2%). In 2013, OXA-48-like carbapenemase was observed for the first time in this neonatal unit, prompting a thorough investigation of these isolates.

### Detailed characterization of OXA-48-like carbapenemase-producing strains.

All the OXA-48-like producers were Klebsiella pneumoniae (*Kp1* to *Kp11*). Some of the neonates from whom the K. pneumoniae was isolated did not survive, and most were “outborns” referred from some other hospitals (data not shown).

*Kp1* to *Kp11* were resistant to most of the antimicrobials tested, *viz.*, piperacillin and its inhibitor (tazobactam), amikacin or gentamicin, cefotaxime, cefoxitin, ciprofloxacin, imipenem, ertapenem, meropenem, and aztreonam, and were fully susceptible to tigecycline (Table 1), although few strains were susceptible to meropenem and cefoxitin.

**TABLE 1:** Susceptibility profiles of K. pneumoniae strains and their transconjugants (TCs)/transformants (TFs) along with transmissibility of *bla*_OXA-181-like_ and genotypic characterization of TCs/TFs established with PCR-based techniques

Strain ID	MIC (mg/liter):^*a*^													Resistance genes present/transferred^*b*^	Insertion sequence (IS) element	Plasmid type
	AN	CN	AT	CT	FX	CI	IP	ETP	MP	CO	TGC	PP	PTZ			
EN5153 (*Kp1*)	8	96	>256	>256	48	4	>32	>32	12	1	0.75	>256	96	*bla*_CTX-M-15_, *bla*_TEM-1B_, *bla*_SHV-1_, *bla*_OXA-1_, *bla*_OXA-181_, *oqxA, oqxB, aac(6′)-Ib-cr*	Truncated IS*Ecp1* (167 bp)	IncFIIK, IncFIB(K), IncFIB (pQil), ColKP3
*Kp1* TF2	3	0.5	0.094	0.38	12	<0.002	8	1	0.38	0.25	0.125	128	48	***bla*_OXA-181_**	ND	ColKP3
EN5172 (*Kp2*)	>256	>1,024	48	>256	6	32	16	>32	2	1	0.38	>256	>256	*bla*_CTX-M-15_, *bla*_TEM-1B_, *bla*_OXA-1_, *bla*_OXA-181_, *aac(6′)-Ib*, *qnrB*, *oqxA*, *oqxB*, *aac(6′)-Ib-cr*	IS*Ecp1* absent but 303 bp of its RTE Ext present	IncFIIK, IncFII, ColKP3
*Kp2* TF2	4	0.5	2	2	6	<0.002	4	12	1	0.5	0.125	128	96	***bla*_OXA-181_**	ND	ColKP3
*Kp2* TF3	2	0.38	0.19	0.5	4	<0.002	4	6	1	0.125	0.125	128	96	*bla*_TEM-1B_, ***bla*_OXA-181_**	ND	ColKP3, IncFII
EN5199 (*Kp3*)	>256	>1,024	>256	>256	>256	>32	16	>32	>32	1	1	>256	>256	*bla*_CTX-M-15_, *bla*_TEM-1B_, *bla*_SHV-28_, *bla*_OXA-1_, *bla*_OXA-9_, *bla*_NDM-5_, *bla*_OXA-232_, *rmtB, aac(6′)-Ib*, *oqxA*, *oqxB*, *aac(6′)-Ib-cr*	IS*Ecp1* absent but 335 bp of its RTE Ext present	IncR, IncFIIK, IncFII, IncFIB (K), IncFIA (HI1), ColKP3
*Kp3* TC1	>256	>1024	16	96	48	>32	>32	32	24	0.5	0.19	>256	>256	***bla*_NDM-5_**, *rmtB*	ND	IncFII
*Kp3* TC4	>256	>1,024	16	96	48	>32	>32	32	24	0.5	0.75	>256	>256	*bla*_CTX-M-15_, *bla*_TEM-1B_, ***bla*_NDM-5_**, ***bla*_OXA-232_**, *rmtB*	ND	ColKP3, IncR, IncFII
EN5213 (*Kp4*)	>256	>1,024	>256	>256	12	>32	16	>32	3	1	1	>256	>256	*bla*_CTX-M-15_, *bla*_SHV-28_, *bla*_OXA-1_, *bla*_OXA-181_, *aac(6′)-Ib*, *qnrB*, *oqxA*, *oqxB*, *aac(6′)-Ib-cr*	IS*Ecp1* absent but 339 bp of its RTE Ext present	IncFIIK, ColKP3
*Kp4 TF1*	2	0.25	0.19	0.75	3	<0.002	16	32	3	0.125	0.19	>256	>256	***bla*_OXA-181_**	ND	ColKP3
EN5218 (*Kp5*)	32	2	32	256	4	>32	8	>32	1	1	1	>256	>256	*bla*_CTX-M-15_, *bla*_TEM-1A_, *bla*_SHV-28_, *bla*_OXA-1_, *bla*_OXA-9_, *bla*_OXA-181_, *aac(6′)-Ib*, *qnrB1*, *oqxA*, *oqxB*, *aac(6′)-Ib-cr*	NF	IncFIIK, IncFII
*Kp5* TC2	32	3	32	32	1	>32	4	0.5	0.032	0.5	1	>256	>256	*bla*_CTX-M-15_, *bla*_SHV-28_, ***bla*_OXA-181_**, *aac(6′)-Ib-cr*, *qnrB*, *oqxA*, *aac(6′)-Ib-cr*	ND	IncFIIK
EN5275 (*Kp6*)	>256	>1,024	>256	>256	>256	>32	32	>32	>32	0.25	0.5	>256	>256	*bla*_CTX-M-15_, *bla*_TEM-1B_, *bla*_SHV-190_, *bla*_CMY-4_, *bla*_OXA-232_, *armA, rmtF, aac(6′)-Ib-Hangzhou*, *qnrB1*, *oqxA*, *oqxB*, *aac(6′)-Ib-cr*	IS*Ecp1* absent but ∼128 bp of its RTE Ext present	Col4401I, Col4401II, ColKP3, IncA/C2, IncFIIK, IncX3, IncFIB(pQil)
*Kp6* TF1	2	1.5	0.5	0.5	32	0.006	8	8	1	0.5	1	>256	>256	***bla*_OXA-232_**	ND	ColKP3
EN5280 (*Kp7*)	>256	>1,024	>256	>256	>256	>32	32	>32	>32	0.5	0.38	>256	>256	*bla*_CTX-M-15_, *bla*_SHV-11_, *bla*_CMY-4_, *bla*_OXA-232_, *armA*, *aac(6′)-Ib*, *qnrB1*, *oqxA*, *oqxB*, *aac(6′)-Ib-cr*	IS*Ecp1* absent but 335 bp of its RTE Ext present	IncA/C, IncFIIK, IncX3, ColKP3
*Kp7* TF1	2	0.25	0.125	1	24	<0.002	8	6	1.5	0.125	0.19	>256	>256	***bla*_OXA-232_**	ND	ColKP3
EN5338 (*Kp8*)	>256	>1,024	>256	>256	>256	>32	>32	>32	>32	0.5	1	>256	>256	*bla*_CTX-M-15_, *bla*_TEM-1B_, *bla*_SHV-28_, *bla*_OXA-232_, *rmtF*, *aac(6′)-Ib-Hangzhou*, *oqxA*, *oqxB*, *qnrS1*	IS*Ecp1* absent but ∼320 bp of its RTE Ext present	ColKP3, IncFIA, IncHI1B, IncFIB (Mar), IncFIB (pQil), IncFIIK, IncFII (pAMA1167-NDM-5)
*Kp8* TF1	3	0.25	0.5	0.75	16	0.16	8	16	1.5	<0.25	0.38	>256	>256	***bla*_OXA-232_**	ND	ColKP3
EN5339 (*Kp9*)	>256	>1,024	>256	>256	>256	>32	>32	>32	>32	64	1	>256	>256	*bla*_CTX-M-15_, *bla*_TEM-1A_, *bla*_OXA-1_, *bla*_OXA-9_, *bla*_NDM-5_, *bla*_OXA-181_, *rmtB*, *aac(6′)-Ib*, *oqxA*, *oqxB*, *aac(6′)-Ib-cr*	IS*Ecp1* absent but 335 bp of its RTE Ext present	ColKP3, IncFIA (HI1), IncFIB (K), IncFIB (pKPHS1), IncR, IncFIIK, IncFII
*Kp9* TC2	>256	>1,024	32	>32	64	>32	>32	>32	6	0.5	1	>256	>256	***bla*_NDM-5_**, *rmtB*, *oqxA*, *oqxB*	ND	IncFII
*Kp9* TC3	>256	>1,024	32	>32	96	>32	>32	>32	12	0.5	1	>256	>256	***bla*_NDM-5_**, ***bla*_OXA-181_**, *rmtB*, *oqxA*, *oqxB*	ND	IncR, IncFII, ColKP3
EN5340 (*Kp10*)	>256	>1,024	>256	>256	>256	>32	>32	>32	>32	64	0.75	>256	>256	*bla*_CTX-M-15_, *bla*_TEM-1A_, *bla*_OXA-1_, *bla*_OXA-9_, *bla*_NDM-5_, *bla*_OXA-181_, *rmtB*, *aac(6′)-Ib*, *oqxA*, *oqxB*, *aac(6′)-Ib-cr*	IS*Ecp1* absent but 335 bp of its RTE Ext present	ColKP3, IncFIA (HI1), IncFIB, IncR, IncFIIK, IncFII
*Kp10* TC1	>256	>1,024	48	>32	96	>32	>32	24	32	0.25	1	>256	>256	***bla*_NDM-5_, *bla*_OXA-181_**, *oqxA*, *oqxB*	ND	ColKP3, IncFII, IncR
EN5343 (*Kp11*)	>256	>1,024	>256	>256	>256	>32	>32	>32	>32	64	1	>256	>256	*bla*_CTX-M-15_, *bla*_TEM-1A_, *bla*_OXA-1_, *bla*_OXA-9_, *bla*_NDM-5_, *bla*_OXA-181_, *rmtB*, *aac(6′)-Ib*, *oqxA*, *oqxB*, *aac(6′)-Ib-cr*	IS*Ecp1* absent but 335 bp of its RTE Ext present	ColKP3, IncFIA (HI1), IncFIB, IncR, IncFIIK, IncFII
*Kp11* TC1	>256	>1,024	32	>32	32	>32	>32	24	>32	0.5	0.25	>256	>256	***bla*_NDM-5_**, *rmtB*, *oqxA*, *oqxB*	ND	IncFII
*Kp11* TC2	>256	>1,024	48	>32	48	>32	>32	12	24	0.5	1	>256	>256	***bla*_NDM-5,_*bla*_OXA-181_**, *rmtB*, *oqxA*, *oqxB*	ND	ColKP3, IncR, IncFII

a Abbreviations: TC, transconjugant; TF, transformant; AN, amikacin; CN, gentamicin; AT, aztreonam; CT, cefotaxime; FX, cefoxitin; CI, ciprofloxacin; IP, imipenem; ETP, ertapenem; MP, meropenem; CO, colistin; TGC, tigecycline; PP, piperacillin; PTZ, piperacillin-tazobactam; ND, not done; NF, not found; RTE Ext, right-end extremity of IS*Ecp1*.

b Transferred carbapenem-resistant genes have been boldfaced.

Two types of OXA-48-like carbapenemases namely, *bla*_OXA-181_ and *bla*_OXA-232_, were found among the study strains, henceforth called *bla*_OXA-181-like_. *bla*_NDM-5_ was the only class B carbapenemase detected and was found in four of the *bla*_OXA-181-like_ positive strains. All 11 *bla*_OXA-181-like_ strains harbor *bla*_CTX-M-15_ along with different β-lactamases and aminoglycoside resistance and quinolone resistance genes in various combinations (Table 2).

**TABLE 2:** Characterization and comparative analysis of the strains by two different methods, *viz*., PCR and whole-genome sequencing (WGS)^*a*^

Strain characteristics			PCR-based findings						WGS-based findings								
Strain ID	Year of isolation	ST/CC^*b*^	Aminoglycoside resistance genes	Beta-lactamases and carbapenemases (*bla*)	Quinolone resistance genes	Virulence determinants	PBRT and primer walking	Integron/integrase/GC array	Aminoglycoside resistance genes	Beta-lactamases (*bla*)	Carbapenemases (*bla*)	Quinolone resistance genes	Other resistance genes (family)	Virulence determinants; CPS cluster genes; capsular type; virulence sequence type; integrative conjugative element	Plasmid type	Integron/GC array	GenBank accession no.
*Kp1*	2013	ST48/CC48	Not found	CTX-M-15, TEM-1B, SHV-1, OXA-1, OXA-181	*oqxAB*, *aac(6′)-Ib-cr*	*wabG*, *uge*, *fimH*	IncFIIK, Col	*intI1*	*aac(3)-IIa, aph(6)-Id, aph(3″)-Ib, aadA2*	TEM-1B SHV-1, OXA-1, CTX-M-15	OXA-181	*oqxAB*, *aac(6′)-Ib-cr*	*fosA* (fosfomycin); *mph*(A) (macrolide); *catA1*, *catB3* (phenicol); *sul1*, *sul2* (sulphonamide); *dfrA12* (trimethoprim); *arsABCDR* (arsenic), *pcoABCDERS* (copper), *silABCEFGPRS* (silver)	*mrkABCDFHIJ*; *fimABCDEFGHIK*; *iutA*; *entABCDEFS*, *fepABCDG*, *fes*; *iroEN*; *fyuA*, *irp1*, *irp2*, *ybtAEPQSTUX; rcsA*, *rcsB*; T6SS-I/II/III; LPS *rfb* locus, *wzi62*, *wzc62*; K62, O1/O2v1; ybt14; ICE*Kp5*	IncFIIK, IncFIB (K), IncFIB (pQil), ColKP3	In27	VSLB00000000
*Kp2*	2014	ST48/CC48	*aac(6′)-Ib*	CTX-M-15, TEM-1B, OXA-1, OXA-181	*qnrB*, *oqxAB*, *aac(6′)-Ib-cr*	*wabG*, *uge*, *fimH*	IncFII, IncFIIK, Col	In27, *intI1*	WGS ND	WGS ND	WGS ND	WGS ND	WGS ND	WGS ND	WGS ND	WGS ND	WGS ND
*Kp3*	2014	ST14/CC15	*rmtB*, *aac(6′)-Ib*	NDM-5, CTX-M-15, TEM-1B, SHV-28, OXA-1, OXA-232	*oqxAB*, *aac(6′)-Ib-cr*	*wabG*, *uge*, *fimH*, *mrkD*	IncFII, IncFIIK, IncR, Col	*intI1*	*rmtB*, *aac(6′)-Ib*, *aadA1*, *aadA2*, *aph(6)-Id*, *aph(3″)-Ib*, *aac(3)-IId*	TEM-1B SHV-28 OXA-1,9, CTX-M-15	NDM-5, OXA-232	*oqxAB*, *aac(6′)-Ib-cr*	*fosA* (fosfomycin); *mph*(A), *ere*(A), *erm*(B) (macrolide); *catB3*, *cmlA1, catA1* (phenicol); *sul1*, *sul2* (sulphonamide); *dfrA1*, *dfrA12* (trimethoprim); *arsABCDR* (arsenic); *pcoABCDER* (copper); *silACEFGPRS* (silver); *merACDEPRT* (mercury)	*mrkABCDFHIJ*; *fimABCDEFGHIK*, *pilW*; *iutA*; *entABCDEFS*, *fepABCDG*, *fes*; *iroEN; fyuA*, *irp1*, *irp2*; *ybtAEPQSTUX*; *rcsA*, *rcsB*; T6SS-I/II/III; LPS *rfb* locus, *wzi2*, *wzc2*; K2, O1/O2v1; ybt14; ICE*Kp5*	IncR, IncFIIK, IncFII, IncFIB (K), IncFIA (HI1), ColKP3	In27, In578	VSLC00000000
*Kp4*	2015	ST15/CC15	*aac(6′)-Ib*	CTX-M-15, SHV-28, OXA-1, OXA-181	*qnrB*, *oqxAB*, *aac(6′)-Ib-cr*	*wabG*, *uge, fimH*, *mrkD*	IncFIIK, Col	*intI1*	WGS ND	WGS ND	WGS ND	WGS ND	WGS ND	WGS ND	WGS ND	WGS ND	WGS ND
*Kp5*	2015	ST15/CC15	*aac(6′)-Ib*	CTX-M-15, TEM-1A, SHV-28, OXA-1, OXA-181	*qnrB*, *oqxAB*, *aac(6′)-Ib-cr*	*uge*, *fimH*, *mrkD*	IncFII, IncFIIK	*intI1*	*aac(6′)-Ib*, *aadA1, aph(3″)-Ib*, *aph(6)-Id*, *aac(6′)-Ib3*	TEM-1A SHV-28 OXA-1,9, CTX-M-15	OXA-181	qnrB1, *oqxAB*, *aac(6′)-Ib-cr*	*fosA* (fosfomycin); *mph*(A) (macrolide); *catB3* (phenicol); *sul2* (sulphonamide); *tet*(A) (tetracycline); *dfrA14* (trimethoprim); *arsABCR* (arsenic); *pcoABCDERS* (copper); *silABCEFGPRS* (silver)	*mrkCDH*; *fimCDHK*; *iutA*; *entCEFS*, *fepABCDG*, *fes*; *iroE*; *fyuA*, *irp1*, *irp2*; *ybtAEPQSUX*; *rcsA*; T6SS-I/II/III; LPS *rfb* locus, *wzi151*; K48, O1v1; ybt1; ICE*Kp4*	IncFIIK, IncFII	In191	WMCH00000000
*Kp6*	2016	ST23/CC23	*armA*, *aac(6′)-Ib*	CTX-M-15, TEM-1B, SHV-190, OXA-232, CMY-4	*qnrB*, *oqxAB*, *aac(6′)-Ib-cr*	*wabG*, *uge*, *fimH*, *mrkD*, *kfuBC*, *wcaJ*, *rmpA*, *magA*	IncA/C, IncFIIK, IncX3, Col	*intI1*	*armA*, *rmtF* *aac(6′)-Ib*, *aph(3″)-Ib*, *aph(6)-Id*	TEM-1B SHV-190, CTX-M-15, CMY-4	OXA-232	qnrB1, *oqxAB*, *aac(6′)-Ib-cr*	*fosA* (fosfomycin); *msr*(E), *mph*(E) (macrolide); *catA1* (phenicol); *arr-2* (rifampin); *sul1*, *sul2* (sulphonamide); *dfrA14* (trimethoprim); *pcoABCDER* (copper); *silCERS* (silver); *terABDEWZ* (tellurite); *pbrAR* (lead)	*mrkABCDFIJ*; *fimABCDEFGHIK*; *iutA*, *iucABCD*; *entABCDEFS*, *fepABCDG*, *fes*; *iroBCDEN*; *fyuA*, *irp1*, *irp2*; *ybtAEPQSTUX*; *allABCDRS*; *rmpA*, *rmpA2*, *magA*; *rcsA*, *rcsB*; T6SS-I/II/III; LPS *rfb* locus, *wzi1*, *wzc1*; K1, O1v2; ybt9; ICE*Kp3*	Col4401I, Col4401II, ColKP3, IncA/C2, IncFIIK, IncX3, IncFIB (pQil)	*aacA4*, *arr-2*, *dfrA14b*	VINI00000000
*Kp7*	2016	ST23/CC23	*armA*, *aac(6′)-Ib*	CTX-M-15, TEM-1B, SHV-11, OXA-232, CMY-4	*qnrB, oqxAB*, *aac(6′)-Ib-cr*	*wabG*, *uge, fimH, mrkD*, *kfuBC*, *wcaJ*	IncA/C, IncFIIK, IncX3, Col	*intI1/aadA2*, *dfrA12*, *orfF*	WGS ND	WGS ND	WGS ND	WGS ND	WGS ND	WGS ND	WGS ND	WGS ND	WGS ND
*Kp8*	2016	ST231/CC231	*aac(6′)-Ib*	CTX-M-15, TEM-1B, SHV-28, OXA-232	*qnrS*, *oqxAB*	*wabG*, *uge, fimH, mrkD*, *kfuBC*	IncFIIK, IncFIA, IncFIB-M, IncHIB-M, Col	In27/*intI1*	*rmtF*, *aac(6′)-Ib, aac(6′)-Ib-Hangzhou*, *aadA2*	TEM-1B SHV-28, CTX-M-15	OXA-232	*qnrS1* *oqxAB*	*fosA* (fosfomycin); *mph*(A) *erm*(B) (macrolide); *catA1* (phenicol); *arr-2* (rifampin); *sul1* (sulphonamide); *dfrA12* (trimethoprim);*terABCDEXZ* (tellurite)	*mrkABCDFHIJ*; *fimABCDEFGHIK*, *pilW*; *iucABCD*, *iutA*; *entABCDEFS*, *fepABCDG*, *fes*; *iroEN*; *fyuA*, *irp1*, *irp2*, *ybtAEPQSTUX*; *sitC*, *sitABCD*; *rcsA*, *rcsB*; T6SS-I/II/III; LPS *rfb* locus; *stbABCDE*; *wzi104*; KL51, O1v2; ybt14; ICE*Kp5*	ColKP3, IncFIA, IncHI1B, IncFIB (Mar), IncFIB (pQil), IncFIIK, IncFII (pAMA1167-NDM-5)	In27, In406	JAAGUA000000000
*Kp9*	2016	ST14/CC15	*rmtB*, *aac(6′)-Ib*	NDM-5, CTX-M-15, TEM-1A, SHV-28, OXA-1, OXA-181	*oqxAB*, *aac(6′)-Ib-cr*	*wabG*, *uge, fimH*, *mrkD*, *kfuBC*	IncFII, IncFIIK, IncR, Col	*intI1*	*rmtB, aac(6′)-Ib*, *aadA1*, *aadA2*, *aph(6)-Id*, *aac(3)-IId, aph(3″)-Ib*	TEM-1A SHV-28 OXA-1,9, CTX-M-15	NDM-5, OXA-181	*oqxAB*, *aac(6′)-Ib-cr*	*fosA* (fosfomycin); *mph*(A), *ere*(A), *erm*(B) (macrolide); *catB3*, *cmlA1, catA1* (phenicol); *sul1*, *sul2* (sulphonamide); *dfrA1*, *dfrA12* (trimethoprim); *arsABCDR* (arsenic); *pcoABCDER* (copper); *silACEFGPRS* (silver); *merACDEPRT* (mercury)	*mrkABCDFHIJ*; *fimABCDEFGHIK*, *pilW*; *iutA*; *entABCDEFS; fepABCDG*, *fes*, *iroEN*, *fyuA*, *irp2*, *ybtAEQSTUX*; *rcsA*, *rcsB*; T6SS-I/II/III; LPS *rfb* locus, *wzi2*, *wzc2*; K2, O1v1; ybt 14; ICE*Kp5*	ColKP3, IncFIA (HI1), IncFIB (K), IncFIB (pKPHS1), IncR, IncFIIK, IncFII	In27, In1329	VSJI00000000
*Kp10*	2016	ST14/CC15	*rmtB*, *aac(6′)-Ib*	NDM-5, CTX-M-15, TEM-1A, SHV-28, OXA-1, OXA-181	*oqxAB*, *aac(6′)-Ib-cr*	*wabG*, *uge, fimH*, *mrkD*, *kfuBC*	IncFII, IncFIIK, IncR, Col	*intI1*	WGS ND	WGS ND	WGS ND	WGS ND	WGS ND	WGS ND	WGS ND	WGS ND	WGS ND
*Kp11*	2016	ST14/CC15	*rmtB*, *aac(6′)-Ib*	NDM-5, CTX-M-15, TEM-1A, SHV-28, OXA-1, OXA-181	*oqxAB*, *aac(6′)-Ib-cr*	*wabG*, *uge, fimH*, *mrkD*, *kfuBC*	IncFII, IncFIIK, IncR, Col	*intI1*	WGS ND	WGS ND	WGS ND	WGS ND	WGS ND	WGS ND	WGS ND	WGS ND	WGS ND

a Terms used: Inc, incompatibility group; *intI1*, integrase 1; GC, gene cassette; *mrk*, type-3 fimbriae operon; *fim*, type-1 fimbriae operon; *pil*, type IV pili; *iuc* and *iut*, aerobactin; *ent*, *fep*, and *fes*, enterobactin; *iro*, salmochelin; *all*, allantoin utilization/nutritional factor; *sitC*, ferrous iron transporter; *sit*, iron/manganese transporter; *rcs*, RcsAB operon; T6SS, type-6-secretion system; LPS *rfb* locus, for serum resistance; *fyuA*, *irp*, and *ybt*, yersiniabactin; *stb*, fimbrae adherence determinants; YbST, yersiniabactin sequence type; ICE, integrative conjugative element; WGS ND, whole-genome sequencing not done.

b ST, sequence type; CC, clonal complex.

### Molecular typing of OXA-48-like carbapenemase-producing strains.

**FIG 1:**
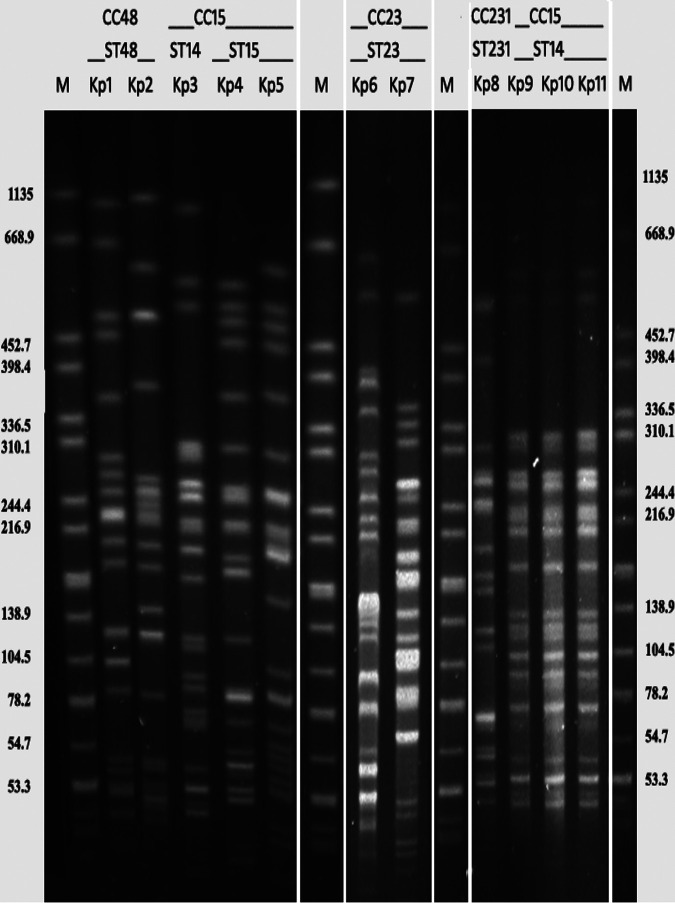
Pulsed-field gel electrophoresis with XbaI macrodigestion of *bla*_OXA-181-like_-harboring K. pneumoniae isolated from blood of septicemic neonates. Lanes 1, 7, 10, and 15: M (marker) *Salmonella* serotype Braenderup H9812 as reference standard; lane 2: *Kp1*; lane 3: *Kp2*; lane 4: *Kp3*; lane 5: *Kp4*; lane 6: *Kp5*; lane 8: *Kp6*; lane 9: *Kp7*; lane 11: *Kp8*; lane 12: *Kp9*; lane 13: *Kp10*; lane 14: *Kp11*. Sequence types found in the strains are listed above each strain. ST, sequence type; CC, clonal complexes.

Pulsed-field gel electrophoresis (PFGE) revealed 7 pulsotypes among the 11 *bla*_OXA-181-like_ K. pneumoniae isolates. Of them, *Kp3* and *Kp9* to *Kp11* were found to be clonal (Fig. 1).

Multilocus sequence typing (MLST) revealed the presence of 5 diverse STs, *viz*., ST14 (*Kp3*, *Kp9* to *Kp11*), ST15 (*Kp4*, *Kp5*), ST23 (*Kp6*, *Kp7*), ST48 (*Kp1*, *Kp2*), and ST231 (*Kp8*) (Table 2). Though *Kp3* and *Kp9* to *Kp11* belonged to same pulsotype and were ST14, their isolation was temporally distant, i.e., *Kp3* in 2014 but *Kp9* to *Kp11* in 2016. They also harbor two different variants of OXA-48-like carbapenemases, *viz*., *bla*_OXA-232_ (*Kp3*) and *bla*_OXA-181_ (*Kp9* to *Kp11*).

The 5 STs collate within 4 clonal complexes (CCs), CC15 (ST14 and ST15), CC23 (ST23), CC48 (ST48), and CC231 (ST231) by goeBURST (Table 2). ST15, ST23, ST48, and ST231 of this study are the founder STs of their respective CCs, harboring the largest number of single-locus variants (SLVs) in their group. ST15, being a single-locus variant of ST14, contains more SLVs than ST14 and has been assigned as the founder of CC15. Hence, ST14 is categorized under CC15 as a subgroup founder. In our study, the presence of *bla*_OXA-181_ was found in ST14, ST15, and ST48, while *bla*_OXA-232_ was found in ST14, ST23, and ST231 (Table 2). On the other hand, *bla*_NDM-5_ was found in ST14 only.

### Resistome and virulome analysis of OXA-181-like carbapenemase-producing strains.

One strain from each different ST (ST15, ST23, ST48, and ST231) along with the 2 strains of ST14 (*Kp3* and *Kp9*) possessing different *bla*_OXA-181-like_ genes were subjected to whole-genome sequencing (WGS). Other strains (*Kp2*, *Kp4*, *Kp7*, *Kp10*, and *Kp11*) not processed for WGS were screened by PCR followed by Sanger sequencing of the relevant resistance genes. Resistome analysis (≥98% identity and coverage) showed the presence of *bla*_CTX-M-15_ in all the strains together with several other β-lactamases, aminoglycoside, and fluoroquinolones (Table 2). Apart from these, the presence of several heavy metal and other antibiotic resistance genes was also noted, as listed in Table 2. Out of 11, 7 were found to carry *bla*_OXA-181_ (*Kp1*, *Kp2*, *Kp4*, *Kp5*, and *Kp9* to *Kp11*), and the remaining 4 (*Kp3*, *Kp6* to *Kp8*) harbored *bla*_OXA-232_.

Strains were found to possess virulence genes (Table 2) such as *iut*, *ent*, *fep*, *fes*, *ybt*, *irp*, *iro*, etc. (iron-chelators). The occurrence of serum resistance and antiphagocytosis capsular factors along with different K- and O-loci were found in the strains. Strains also possessed various integrative conjugative elements. The presence of *rmpA*, *rmpA2*, and *magA* responsible for hypermucoidy and hypervirulence was found in *Kp6*, which has already been reported in a separate study (16).

### Transmissibility of *bla*_OXA-181_, *bla*_OXA-232_, and *bla*_NDM-5_.

Conjugal transfer of an OXA-48-like-bearing plasmid was successful for 5 strains (*Kp3*, *Kp5*, and *Kp9* to *Kp11*); for others, transformants were obtained. The presence of resistance genes was assessed in the transconjugants (TCs)/transformants (TFs) (Table 1). *bla*_NDM-5_ was borne on large conjugative plasmids (ranging between ∼100 and 200 kb), while *bla*_OXA-181/OXA-232_ were present on small (∼6 to 8 kb) nonconjugative plasmids. Interestingly, conjugal transfer of *bla*_OXA-181/OXA-232_ was successful when coexisting with *bla*_NDM-5_, though on separate plasmids.

Most of the TCs/TFs with only *bla*_OXA-181-like_ showed the presence of similar plasmid scaffolds, i.e., ColKP3, except for one (*Kp5*) with IncFIIK (Table 1). WGS data also specified the association of ColKP3 with the *bla*_OXA-181-like_ (Table 2). On the other hand, *bla*_NDM-5_ was present on IncFII (Table 1).

The MIC of the TCs/TFs for different antimicrobials were assessed (Table 1). TCs/TFs with only *bla*_OXA-181-like_ exhibited high MICs for imipenem followed by ertapenem compared to meropenem. However, TCs where coexistence of *bla*_NDM-5_ and *bla*_OXA-181-like_ were observed showed higher MIC for meropenem.

### Analysis of mobile genetic elements (MGEs).

**FIG 2:**
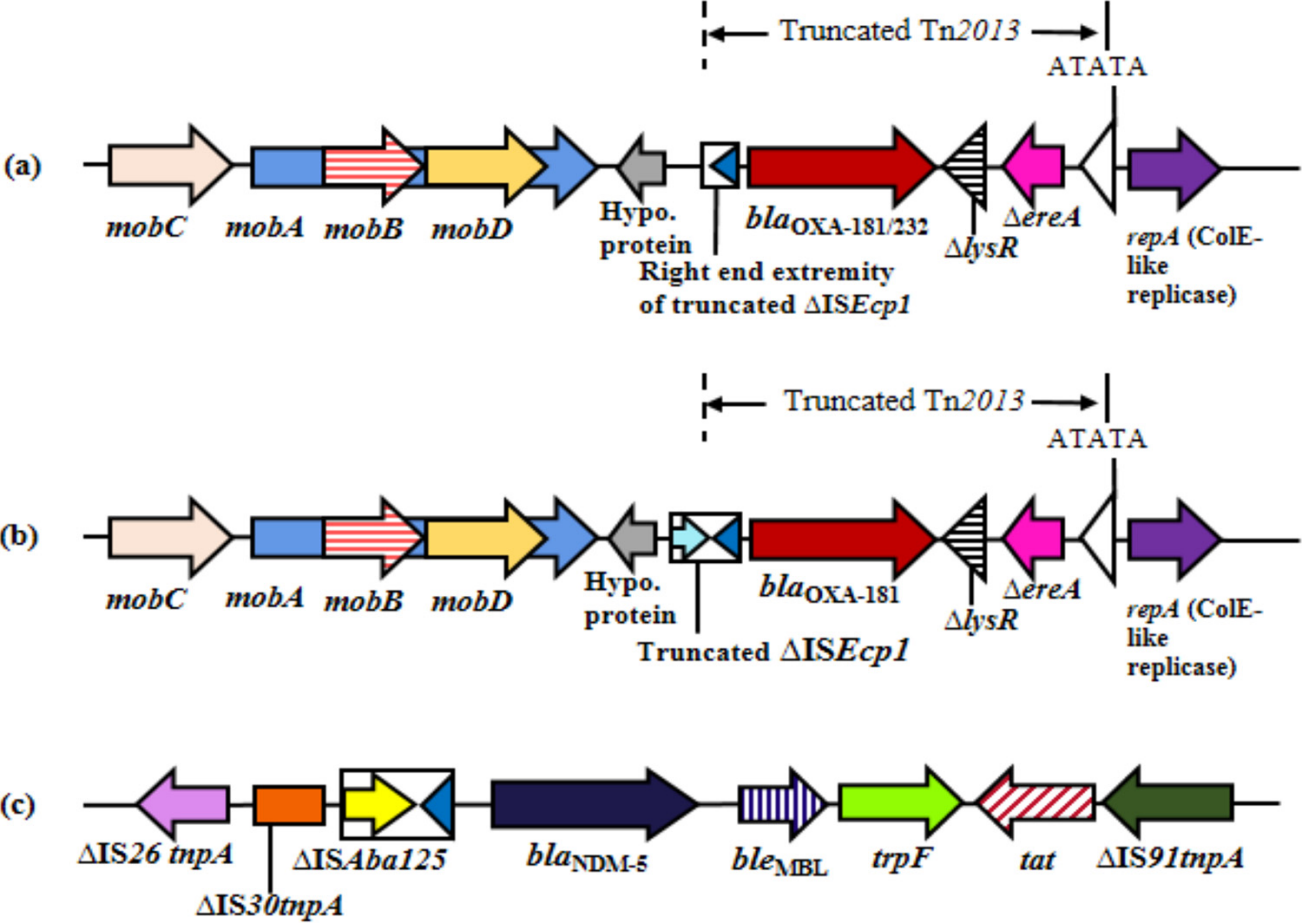
Schematic presentation of MGEs associated with *bla*_OXA-181/232_ and *bla*_NDM-5_ in the K. pneumoniae strains isolated from neonates. Heterogeneity of the genetic environment found in the studied carbapenemases: (a) *bla*_OXA-181/232_ (*Kp2-Kp4*, *Kp6-Kp11*), (b) *bla*_OXA-181_ in *Kp1*, and (c) genetic environment of transposon 125 (Tn*125*) harboring *bla*_NDM-5_ (*Kp3*, *Kp9-Kp11*). Genes and their corresponding transcription orientations are indicated by horizontal arrows. Target site duplications (ATATA) generated by the insertion of Tn*2013* are indicated by white triangles. *mobA*, *mobB*, *mobC*, and *mobD*, mobilization relaxosome proteins; ***Δ****lysR*, truncated LysR-type transcriptional regulator; ***Δ****ereA*, truncated erythromycin esterase; *repA*, replicase; *tnpA*, transposase; IS, insertion sequence; *ble*_MBL_; bleomycin resistance gene; *trpF*, N-(5′-phosphoribosyl) anthranilate isomerase; *tat*, twin-arginine translocation pathway signal sequence protein; Hypo. protein, hypothetical protein; Δ, denotes deletion or truncation.

The genetic environment of *bla*_OXA-181-like_ revealed the presence of a mobilization relaxosome (*mobA*, *mobB*, *mobC*, and *mobD*) upstream, and *ΔlysR* (transcription regulator), *ΔereA* (erythromycin esterase), and Col replicase (*repA*) downstream, respectively (Fig. 2a and b). Deletion of IS*Ecp1* was found with varying stretches of its right-end extremity except for *Kp1* and *Kp5* (Table 1). All study strains were found in truncated Tn*2013*.

On the other hand, *bla*_NDM-5_ was bracketed between truncated IS*Aba125* and bleomycin resistance genes (*ble*_MBL_) found upstream and downstream, respectively. IS*Aba125* is preceded by a truncated transposase of the IS*30* family and truncated IS*26*, while *ble*_MBL_ is succeeded by N-(5′-phosphoribosyl) anthranilate isomerase (*trpF*), twin-arginine translocation pathway signal sequence protein (*tat*), and the truncated transposase of IS*91* (Fig. 2c). *Kp3* and *Kp9-Kp11* have similar genetic environments with truncated Tn*125*.

**FIG 3:**
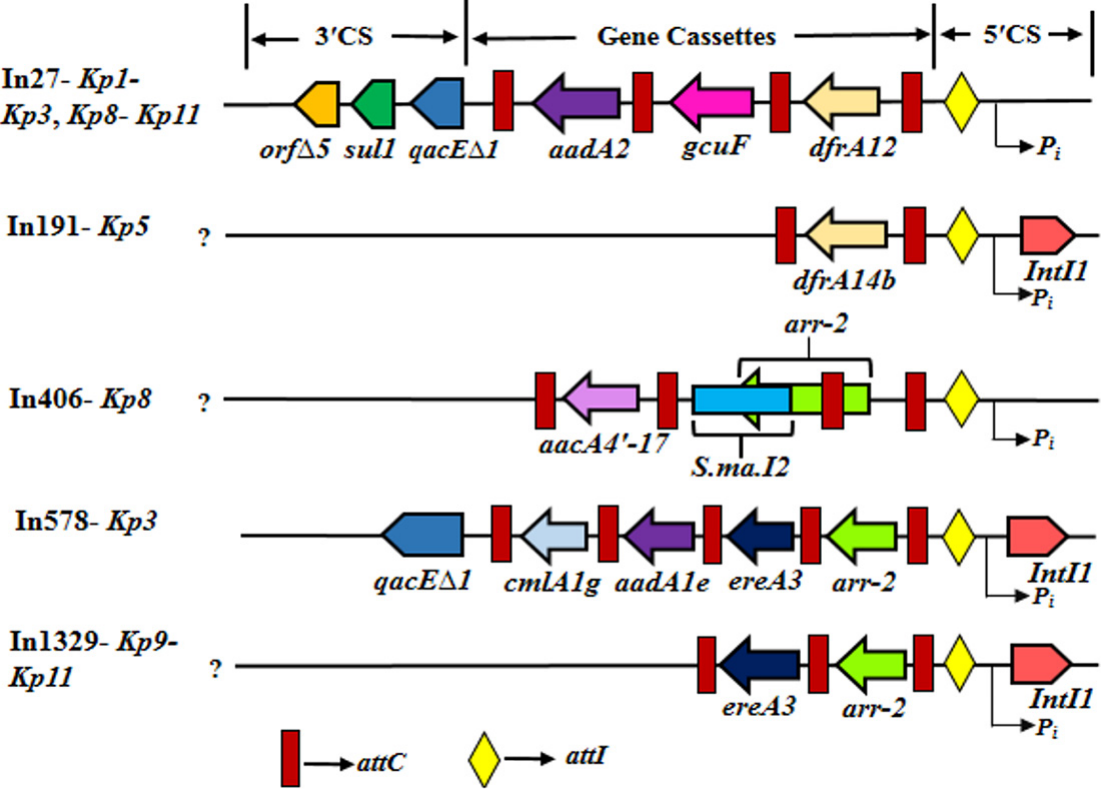
Diagrammatic representation of class 1 integron found in the strains under study. *arr-2*, ADP-ribosyl transferase; *qacEΔ1*, quaternary ammonium compound resistance protein; *sul1*, sulfonamide resistant dihydropteroate synthase; orfΔ5, an open reading frame of unknown function; *aadA1e* and *aadA2*, aminoglycoside adenyltransferase; *gcuF*, DUF1010 domain-containing protein; *dfrA12* and *dfrA14b*, dihydrofolate reductases type-A; *S.ma.I2*, group IIc intron; *aacA4′-17*, aminoglycoside 6′-*N*-acetyltransferase; *ereA3*, erythromycin esterase; *cmlA1g*, chloramphenicol resistance gene; *attI*, site of recombination; *intI1*, integrase gene; *attC*, site of attenuation; P_i_, promoter of integrase; CS, conserved sequence.

Five different integrons, In27, In191, In406, In578, and In1329, were detected (Table 2 and Fig. 3). In27 was found to be the most prevalent integron (*Kp1* to *Kp3*, *Kp8* to *Kp11*) (Table 2), but *bla*_OXA-181/OXA-232_ or *bla*_NDM-5_ was not found to be allied to any of the integrons obtained.

### A phylogenetic global comparison of OXA-48-like genomes and K. pneumoniae isolated from neonates.

**FIG 4:**
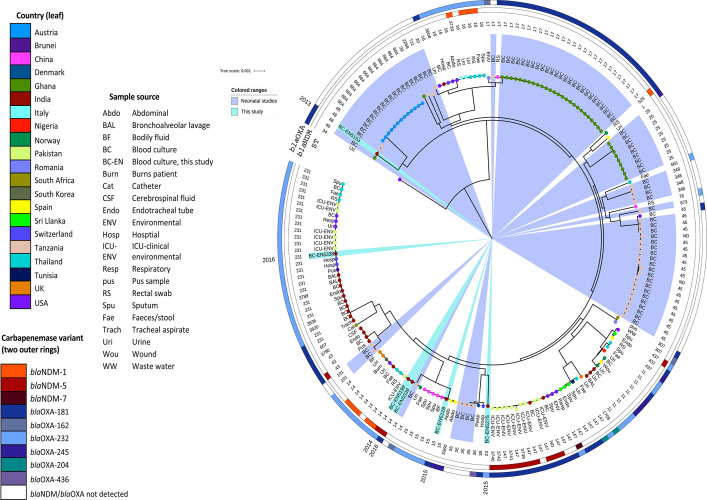
Core genome phylogeny of 197 Klebsiella pneumoniae isolates using Roary (v3.12.0) and FastTree (v2.1.11). Isolates are colored at the endpoint according to country, and the outer ring abbreviation is labeled according to the sample source. The additional two outer rings denote the presence of *bla*_NDM_ and *bla*_OXA-48-like_ antibiotic resistance genes. Clades containing isolates from this study are highlighted in teal, and light blue clade highlights indicate K. pneumoniae neonatal sepsis isolates from other studies. The year of sample collection for isolates in this study has been added external to the tree phylogeny.

The maximum likelihood core genome phylogenetic tree was constructed with 197 K. pneumoniae from (i) a global collection of OXA-48-like and NDM carbapenemase-carrying isolates and (ii) published genomic data of septicemic neonatal K. pneumoniae (Fig. 4). As few neonatal studies with published sequence data (either GenBank NCBI or ENA-EMBL) were available, all possible sequences were incorporated, irrespective of carbapenem resistance.

*bla*_OXA-48-like_ K. pneumoniae detected from 21 countries and 20 sample sources, including human, animal, and environmental samples, were remarkably diverse, with 40 different STs identified.

**FIG 5:**
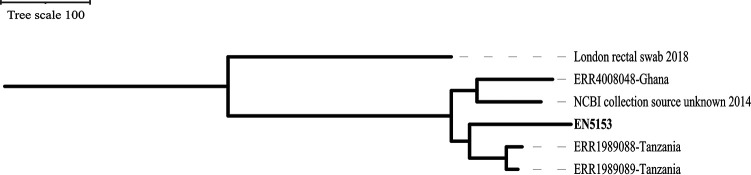
Core genome SNP phylogeny of EN5153 (*Kp1*) with other ST48 neonatal isolates. An outgroup rooted tree was built using the most distant isolate from the Mash genome estimation analysis (an isolate from London, submitted to the NCBI database in 2018). Isolates beginning with ERR are from other ST48 neonatal isolates and another isolate submitted to NCBI on 2014.

The diversity at the core genome level of the strains within this study was vast, spanning multiple lineages, showing both diversity among themselves as causative agents of neonatal sepsis and varying levels of relatedness compared to strains from different parts of the world. EN5153 (*Kp1*) showed similarities with strains from Tanzania and Ghana; EN5218 (*Kp5*), with strains from China, Spain, and Norway; EN5275 (*Kp6*), with distantly related strains from Romania; EN5338 (*Kp8*), with strains from Thailand, Pakistan, the United States, and Switzerland; and EN5199 (*Kp3*) and EN5339 (*Kp9*), with strains from the United Kingdom, the United States, South Korea, Pakistan, Thailand, and Tanzania. Also, EN5199, EN5338, and EN5339 showed similarities with strains reported from various parts of India. When genomes of bacteria causing neonatal infections are compared, EN5153, EN5199, and EN5339 showed similarities with neonatal strains from Tanzania and Ghana. Interestingly, core genome single nucleotide polymorphism (SNP) phylogeny of EN5153 suggests that all ST48 neonatal isolates sit within the same cluster, and the additional ST48 with the greatest similarity from the NCBI database (an isolate from a rectal swab in London from 2018) sits on a single branch (Fig. 5).

Six variants of *bla*_OXA-48-like_ were identified in the collective core genome phylogeny, of which only *bla*_OXA-181_ or *bla*_OXA-232_ were detected from neonatal K. pneumoniae in both Ghana and this study. Apart from these, none of the neonatal strains harbor carbapenem-resistant genes.

## DISCUSSION

In this study, we characterized *bla*_OXA-181-like_-producing K. pneumoniae in a neonatal setting over 4 years, showing the diversity of the genomes. We identified 11 *bla*_OXA-181/232_ carbapenemases-producing K. pneumoniae. *bla*_NDM-5_ was found in some of the strains. OXA-48-like carbapenemases have been found to be the most common carbapenemases among *Enterobacteriaceae* family pathogens in certain parts of the world, such as Europe, the Middle East, North America, etc., while NDM carbapenemases are endemic to India and Southeast Asia (10, 14, 20). The presence of *bla*_OXA-181/OXA-232_ along with *bla*_NDM-5_ has been reported in patients from South Korea, the United States, Chad, and Nepal, having travel history from India or the Indian subcontinent (8, 21–23). The existence of dual carbapenemases (*bla*_OXA-181/232_ and *bla*_NDM-5_) among the strains reduced their susceptibility to all carbapenems (imipenem, ertapenem, and meropenem), thereby making them extremely drug resistant. Infection with these organisms is dreadful, especially in neonates with limited therapeutic options. Following an extensive PubMed search for reports of *bla*_OXA-181/OXA-232_ along with *bla*_NDM-5_ in neonates, we found no matches; however, *bla*_OXA-232_ has been reported in neonatal infections from China (20). Hence, to the best of our knowledge, this is the first study to report the coexistence of *bla*_OXA-181/OXA-232_ with *bla*_NDM-5_ in septicemic neonates.

Strains were found to be diverse and belonged to 5 different STs, some of which are well-known international clones (ST14). OXA-48-like carbapenemases are well known for triggering outbreaks involving specific sequence types, such as ST11, ST14, ST15, ST101, ST147, and ST307 recorded from various parts of Europe, Mediterranean regions, China, North America, and South Africa (12, 14). Carriage of *bla*_OXA-181_ with STs such as ST11, ST14, ST16, ST25, ST43, ST61, ST147, ST231, ST307, and ST709 and *bla*_OXA-232_ with ST11, ST14, ST15, ST16, ST17, ST101, ST147, ST231, ST307, ST395, ST570, and ST2040 have been previously reported (11, 12, 14, 24). Major hospital outbreaks were noted with ST14 and ST15, harboring *bla*_OXA-181_ and *bla*_OXA-232_, respectively, in Canada and China, the latter involving a neonatal unit (14). Reports of *bla*_OXA-181-like_ with ST11, ST14, ST43, ST101, ST147, ST231, and ST2040 were documented from India (11, 24). However, in this study, the occurrence of *bla*_OXA-181_ in ST14, ST15, and ST48 and *bla*_OXA-232_ in ST14, ST23, and ST231 was noted (Table 2). K. pneumoniae isolates with *bla*_NDM-5_ are mostly reported among ST15, ST45, ST147, ST182, ST395, and ST476 (21, 25–28). But the present study, like a few other studies (25, 29), reported *bla*_NDM-5_ in ST14 K. pneumoniae. The presence of *bla*_OXA-181/OXA-232_ with *bla*_NDM-5_ in high-risk international clone ST14 further highlights the spread of resistance across continental boundaries.

A plethora of resistance and virulence genes were identified among the strains, which supports the survival of the pathogen in antibiotic-laden environments of health care settings as well as their successful colonization in the host. The occurrence of resistance genes on plasmids and virulence genes on integrative conjugative elements instigates the spread of these genes in the community. Hence, the presence of drug-resistant virulent strains of K. pneumoniae in neonates can cause severe infection leading to critical consequences.

In the current study, two specific plasmid scaffolds were seen to be associated with the studied carbapenemases genes. *bla*_OXA-181-like_ were found on a nonconjugative ColKP3 plasmid on a truncated Tn*2013*, as reported previously (14, 30, 31). *bla*_OXA-232_ has always been reported in Tn*2013*, but *bla*_OXA-181_ has been found in Tn*2013* and in other transposons, such as Tn*6360* (14). Deletion of IS*Ecp1* from the upstream of *bla*_OXA-181/232_ was noted among the strains, which must have restricted its transposase activity, resulting in stabilization of *bla*_OXA-181/OXA-232_ on pKP3/pOXA232-like plasmids (30, 31). *bla*_NDM-5_ was found in a conjugative IncFII plasmid within truncated Tn*125* with a comparable plasmid background reported from a nontraveler in Spain (32), although the association of *bla*_NDM-5_ is predominantly reported in IncX3, but they have also been found in IncFII (32). This study also indicated the presence of *bla*_OXA-181/OXA-232_ and *bla*_NDM-5_ on separate plasmids, suggesting two independent events of gene acquisition by the organism. The majority of previous reports have proposed that the spread of *bla*_OXA-181-like_ is through clonal dissemination, but this study corroborated the results from few earlier reports (14, 30, 31), describing the involvement of a helper plasmid (*bla*_NDM-5_) that facilitated conjugal transfer of *bla*_OXA-181-like_, reinforcing the role of helper plasmids in their transmission. Such a phenomenon underlines the threat these carbapenemases pose when present with *bla*_NDM_, not only in terms of increased resistance and further treatment limitations, but also in the ease of transfer.

WGS analysis of neonatal strains is largely limited to outbreak cases, and studies of isolates collected over longer periods are rare. This study is probably the first to incorporate a global collection of K. pneumoniae harboring OXA-48-like and NDM carbapenemases with special reference to septicemic neonatal strains. Strains of this study belonged to diverse sequence types, which ruled out clonal spread of *bla*_OXA-181-like_-carbapenemases and were similar to outbreak strains from neonates in Tanzania, Ghana, and Austria (33–35). Genomes were diverse, but the plasmid scaffold (ColKP3) harboring *bla*_OXA-181-like_ was similar across the study strains as also reported by other studies (14, 30, 31). Diversity among the isolates studied here could be, in part, due to many neonatal referrals from other hospitals within this study, and therefore neonates were exposed to both different health care and environmental factors.

Although there are limitations of short read sequencing with respect to plasmid assembly, holistic understanding of the genomes and their spread across the globe and in specific populations or patients is possible. The presence of carbapenem-resistant K. pneumoniae in low-middle-income countries (LMIC) such as India, where neonatal deaths amount to nearly 0.75 million per year (5), is a serious concern which requires rapid investigation. With increasing WGS facilities and decreasing cost of sequencing, short read sequencing is an extremely useful tool to aid routine antimicrobial resistance (AMR) surveillance. This study thus gives an insight about such strains not only in a particular setting but also in a wider global context.

## MATERIALS AND METHODS

### Ethical approval.

The study protocol was approved by the Institutional Ethics Committee of the ICMR-National Institute of Cholera and Enteric Diseases (no. A-1-2/2018/IEC). Patient information was anonymized and deidentified prior to analysis.

### Identification and susceptibility testing.

During 2013 to 2016, bacteria were isolated from blood of septicemic neonates from the neonatal intensive care unit of a tertiary care hospital of Kolkata, West Bengal, India. Isolates were identified with in-house biochemical tests and the Vitek 2 compact system (bioMérieux, Marcy-l’Étoile, France). MICs were determined with Etest (bioMérieux) for all antimicrobials tested, except for colistin. Broth microdilution was carried out for colistin as described previously (36). Results were analyzed according to CLSI and EUCAST guidelines (37, 38).

### Genotypic characterization of β-lactamases, carbapenemases, fluoroquinolones, and 16S rRNA methylases.

PCR was carried out for the following resistance genes: β-lactamase genes (*bla*_CTX-M,TEM,SHV,OXA-1_), AmpC genes (*bla*_MOX,CMY,DHA,ACC,MIR/ACT,FOX_), aminoglycoside resistance genes [*aac(6′)-Ib*, *rmtA*, *rmtB*, *rmtC*, *rmtD*, *and armA*], carbapenemase genes (*bla*_VIM,IMP,SPM-1,GIM-1,SIM-1,NDM-1,_ *bla*_OXA-48,_ *bla*_KPC,SME,IMI,GES,NMC_), and flouroquinolone resistance genes [*qnr-A*,*B*,*S*, *qepA*, *aac(6′)-Ib-cr*, *oqxA*, *oqxB*], depending upon the susceptibility profile (39, 40).

### Multilocus sequence typing (MLST) and pulsed field gel electrophoresis (PFGE).

For sequence typing (ST), seven housekeeping genes were amplified, sequenced, and submitted to the MLST database (https://bigsdb.web.pasteur.fr/cgi-bin/bigsdb/bigsdb.pl?db=pubmlst_klebsiella_seqdef). The goeBURST algorithm (http://www.phyloviz.net/goeburst/) was used for assigning clonal complexes to the STs (41).

Strains producing *bla*_OXA-181-like_ were subjected to PFGE using XbaI and were visually interpreted according to Tenover criteria (42).

### Transmissibility of carbapenem-resistant genes.

Transfer of carbapenemase genes was performed by conjugation with the E. coli J53 Az^r^ strain as the recipient by the solid-mating conjugation technique. Electroporation was carried out with purified plasmid DNA (43) into E. coli DH10B (Invitrogen, California, USA) using a Gene Pulser II (Bio-Rad Laboratories, Hercules, CA, USA) for every failed conjugation. Transconjugants (TCs) were selected on Luria Bertani (LB) agar plates supplemented with (i) sodium azide (100 mg/liter) and ertapenem (0.25 mg/liter) and (ii) sodium azide and cefoxitin (8 mg/liter) (Sigma-Aldrich, St. Louis, MO, USA) for *bla*_OXA-181-like_-producing strains possessing *bla*_NDM_. Transformants (TFs) were selected on LB agar with ertapenem (0.25 mg/liter). The TCs/TFs retrieved were subjected to confirmation of carbapenem-resistant genes and other β-lactamase genes by PCR followed by susceptibility testing.

Plasmid analysis was performed with wild-type strains and their TCs/TFs according to Kado and Liu (43), followed by plasmid typing using PCR-based replicon typing (PBRT) (44). To map the entire integron structure and determine their types and possible association with carbapenem-resistant genes, PCRs were performed as described previously (45, 46), followed by Sanger sequencing, and submitted to the INTEGRALL site.

### Whole-genome sequencing (WGS).

Total genomic DNA was isolated and DNA libraries were prepared for paired-end sequencing using Nextera XT and NEBNext Ultra II DNA library prep kits according to the manufacturer’s instruction. Sequencing was performed using the Illumina platform (San Diego, CA). Quality and adaptor trimming were completed using Trim Galore (v0.4.3). *De novo* assembly was accomplished using different assemblers, such as SPAdes (v.3.9.0), Velvet (v.1.2.10), and Shovill (v.0.9.0), and Pilon (v1.22) was used on the resulting contigs to correct any mapping errors. Evaluation of assembly metrics and annotation were carried out using Quast (v2.1) and Prokka (v1.12), respectively, and were viewed in Artemis (Sanger, UK) and the SnapGene viewer.

With the contig files, the following online servers were used for analysis: (i) ResFinder (https://cge.cbs.dtu.dk/services/ResFinder/) and pathogenwatch (https://pathogen.watch/) for resistance genes, (ii) PlasmidFinder (https://cge.cbs.dtu.dk/services/PlasmidFinder/) for plasmid types, (iii) the MLST database for sequence typing, (iv) the BIGSdb-Kp database (http://bigsdb.web.pasteur.fr/klebsiella/klebsiella.html) and the virulence factor database (VFDB) (http://www.mgc.ac.cn/VFs/main.htm) for virulence genes and the Kaptive database (https://kaptive-web.erc.monash.edu/) for K- and O-antigen capsular typing, (v) the Integrall site for nomenclature of the integron sequences, (vi) TETyper for identification of transposon type, and (vii) ISfinder for IS elements (https://isfinder.biotoul.fr/).

A core genome phylogeny tree was built using Roary (v3.12.0) and FastTree (v2.1.11) with isolates from this study along with K. pneumoniae possessing different OXA-48-like and NDM variants submitted to National Center for Biotechnology Information (NCBI) database. Initially 8,663 K. pneumoniae genomes were downloaded from NCBI on 27 March 2020. Abricate (v0.9.7) was used to screen the genomes for the presence of OXA-48-like and NDM antibiotic resistance genes. Similarly, *in silico* MLST (v2.17.6) was performed to assign STs. Based on the presence/absence of carbapenemase variants, and ST, a selection of strains was chosen for comparative analysis. From the BioSample database within NCBI, data of the country and source of the isolate were collected, where applicable. Additionally, and following a literature search for studies with neonatal sepsis K. pneumoniae WGS data available, raw sequencing reads were downloaded from the ENA repository. All FASTQ reads were subject to the same quality control (QC) parameters as previously described, assembled using Shovill (v0.9.0), and annotated using Prokka (v1.12). Based on relatedness to other neonatal sepsis isolates in the core genome phylogeny, isolates within the same clade were further analyzed to create a core SNP phylogeny using Snippy, Gubbins (47), and RAxML (48) (GTRCAT model) within the Snpiphy (v0.5.0) pipeline with the default 85% coverage cutoff.

To complement this analysis, a genome estimation of all NCBI genomes (*n* = 8663) compared to the study strains was performed using Mash (v2.0), and isolates with a similarity of >950/1,000 shared hashes were additionally incorporated into this analysis.

### Data availability.

All genome sequences were submitted to the NCBI database with accession numbers VSLB00000000, VSLC00000000, WMCH00000000, VINI00000000, JAAGUA000000000, VSJI00000000 (Table 2).

## References

[1] Ahmad N, Ali SM, Khan AU 2019 Molecular characterization of novel sequence type of carbapenem-resistant New Delhi metallo-β-lactamase-1-producing *Klebsiella pneumoniae* in the neonatal intensive care unit of an Indian hospital. Int J Antimicrob Agents 53:525–529. doi:10.1016/j.ijantimicag.2018.12.005. 30578964

[2] Shane AL, Sánchez PJ, Stoll BJ 2017 Neonatal sepsis. Lancet 390:1770–1780. doi:10.1016/S0140-6736(17)31002-4. 28434651

[3] Effah CY, Sun T, Liu S, Wu Y 2020 *Klebsiella pneumoniae*: an increasing threat to public health. Ann Clin Microbiol Antimicrob 19:1. doi:10.1186/s12941-019-0343-8. 31918737 PMC7050612

[4] Le Doare K, Bielicki J, Heath PT, Sharland M 2015 Systematic review of antibiotic resistance rates among gram-negative bacteria in children with sepsis in resource-limited countries. J Pediatric Infect Dis Soc 4:11–20. doi:10.1093/jpids/piu014. 26407352

[5] Sankar MJ, Neogi SB, Sharma J, Chauhan M, Srivastava R, Prabhakar PK, Khera A, Kumar R, Zodpey S, Paul VK 2016 State of newborn health in India. J Perinatol 36:S3–S8. doi:10.1038/jp.2016.183. PMC5144119 27924104

[6] Poirel L, Potron A, Nordmann P 2012 OXA-48-like carbapenemases: the phantom menace. J Antimicrob Chemother 67:1597–1606. doi:10.1093/jac/dks121. 22499996

[7] Queenan AM, Bush K 2007 Carbapenemases: the versatile beta-lactamases. Clin Microbiol Rev 20:440–458. doi:10.1128/CMR.00001-07. 17630334 PMC1932750

[8] Rojas LJ, Hujer AM, Rudin SD, Wright MS, Domitrovic TN, Marshall SH, Hujer KM, Richter SS, Cober E, Perez F, Adams MD, van Duin D, Bonomo RA 2017 NDM-5 and OXA-181 beta-lactamases, a significant threat continues to spread in the Americas. Antimicrob Agents Chemother 61:e00454-17. doi:10.1128/AAC.00454-17. 28461314 PMC5487671

[9] Hornsey M, Phee L, Wareham DW 2011 A novel variant, NDM-5, of the New Delhi metallo-β-lactamase in a multidrug-resistant *Escherichia coli* ST648 isolate recovered from a patient in the United Kingdom. Antimicrob Agents Chemother 55:5952–5954. doi:10.1128/AAC.05108-11. 21930874 PMC3232805

[10] Wu W, Feng Y, Tang G, Qiao F, McNally A, Zong Z 2019 NDM metallo-β-lactamases and their bacterial producers in health care settings. Clin Microbiol Rev 32:e00115-18. doi:10.1128/CMR.00115-18. 30700432 PMC6431124

[11] Mairi A, Pantel A, Sotto A, Lavigne JP, Touati A 2018 OXA-48-like carbapenemases producing Enterobacteriaceae in different niches. Eur J Clin Microbiol Infect Dis 37:587–604. doi:10.1007/s10096-017-3112-7. 28990132

[12] Findlay J, Hopkins KL, Loy R, Doumith M, Meunier D, Hill R, Pike R, Mustafa N, Livermore DM, Woodford N 2017 OXA-48-like carbapenemases in the UK: an analysis of isolates and cases from 2007 to 2014. J Antimicrob Chemother 72:1340–1349. doi:10.1093/jac/dkx012. 28199647

[13] Al-Baloushi AE, Pál T, Ghazawi A, Sonnevend A 2018 Genetic support of carbapenemases in double carbapenemase producer *Klebsiella pneumoniae* isolated in the Arabian Peninsula. Acta Microbiol Immunol Hung 65:135–150. doi:10.1556/030.65.2018.005. 29471690

[14] Pitout JDD, Peirano G, Kock MM, Strydom KA, Matsumura Y 2019 The global ascendency of OXA-48-type carbapenemases. Clin Microbiol Rev 33:e00102-19. doi:10.1128/CMR.00102-19. 31722889 PMC6860007

[15] Nigg A, Brilhante M, Dazio V, Clément M, Collaud A, Gobeli Brawand S, Willi B, Endimiani A, Schuller S, Perreten V 2019 Shedding of OXA-181 carbapenemase-producing *Escherichia coli* from companion animals after hospitalisation in Switzerland: an outbreak in 2018. Euro Surveill 24:1900071 https://www.eurosurveillance.org/content/10.2807/1560-7917.ES.2019.24.39.1900071. 10.2807/1560-7917.ES.2019.24.39.1900071 PMC6774230 31576806

[16] Mukherjee S, Naha S, Bhadury P, Saha B, Dutta M, Dutta S, Basu S 2020 Emergence of OXA-232-producing hypervirulent *Klebsiella pneumoniae* ST23 causing neonatal sepsis. J Antimicrob Chemother 75:2004–2006. doi:10.1093/jac/dkaa080. 32155265

[17] Castanheira M, Deshpande LM, Mathai D, Bell JM, Jones RN, Mendes RE 2011 Early dissemination of NDM-1- and OXA-181-producing Enterobacteriaceae in Indian hospitals: report from the SENTRY Antimicrobial Surveillance Program, 2006–2007. Antimicrob Agents Chemother 55:1274–1278. doi:10.1128/AAC.01497-10. 21189345 PMC3067112

[18] Fröhlich C, Sørum V, Thomassen AM, Johnsen PJ, Leiros HS, Samuelsen Ø 2019 OXA-48-mediated ceftazidime-avibactam resistance is associated with evolutionary trade-offs. mSphere 4:e00024-19. doi:10.1128/mSphere.00024-19. 30918055 PMC6437269

[19] Bakthavatchalam YD, Anandan S, Veeraraghavan B 2016 Laboratory detection and clinical implication of oxacillinase-48 like carbapenemase: the hidden threat. J Glob Infect Dis 8:41–50. doi:10.4103/0974-777X.176149. 27013843 PMC4785756

[20] Yin D, Dong D, Li K, Zhang L, Liang J, Yang Y, Wu N, Bao Y, Wang C, Hu F 2017 Clonal dissemination of OXA-232 carbapenemase-producing *Klebsiella pneumoniae* in neonates. Antimicrob Agents Chemother 61:e00385-17. doi:10.1128/AAC.00385-17. 28533245 PMC5527636

[21] Sherchan JB, Tada T, Shrestha S, Uchida H, Hishinuma T, Morioka S, Shahi RK, Bhandari S, Twi RT, Kirikae T, Sherchand JB 2020 Emergence of clinical isolates of highly carbapenem-resistant *Klebsiella pneumoniae* co-harboring *bla*_NDM-5_ and *bla*_OXA-181 or -232_ in Nepal. Int J Infect Dis 92:247–252. doi:10.1016/j.ijid.2020.01.040. 31982619

[22] Cho SY, Huh HJ, Baek JY, Chung NY, Ryu JG, Ki CS, Chung DR, Lee NY, Song JH 2015 *Klebsiella pneumoniae* co-producing NDM-5 and OXA-181 carbapenemases, South Korea. Emerg Infect Dis 21:1088–1089. doi:10.3201/eid2106.150048. 25988911 PMC4451906

[23] Ouchar Mahamat O, Lounnas M, Hide M, Tidjani A, Benavides J, Diack A, Somasse C, Gamougam K, Carrière C, Decré D, Bañuls AL, Jean-Pierre H, Dumont Y, Compain F, Godreuil S 2019 Spread of NDM-5 and OXA-181 carbapenemase-producing *Escherichia coli* in Chad. Antimicrob Agents Chemother 63:e00646-19. doi:10.1128/AAC.00646-19. 31405861 PMC6811454

[24] Shankar C, Mathur P, Venkatesan M, Pragasam AK, Anandan S, Khurana S, Veeraraghavan B 2019 Rapidly disseminating *bla*_OXA-232_ carrying *Klebsiella pneumoniae* belonging to ST231 in India: multiple and varied mobile genetic elements. BMC Microbiol 19:137. doi:10.1186/s12866-019-1513-8. 31234800 PMC6591861

[25] Moubareck CA, Mouftah SF, Pál T, Ghazawi A, Halat DH, Nabi A, AlSharhan MA, AlDeesi ZO, Peters CC, Celiloglu H, Sannegowda M, Sarkis DK, Sonnevend Á 2018 Clonal emergence of *Klebsiella pneumoniae* ST14 co-producing OXA-48-type and NDM carbapenemases with high rate of colistin resistance in Dubai, United Arab Emirates. Int J Antimicrob Agents 52:90–95. doi:10.1016/j.ijantimicag.2018.03.003. 29530587

[26] Khalifa HO, Soliman AM, Ahmed AM, Shimamoto T, Shimamoto T 2016 NDM-4- and NDM-5-producing *Klebsiella pneumoniae* coinfection in a 6-month-old infant. Antimicrob Agents Chemother 60:4416–4417. doi:10.1128/AAC.00479-16. 27185797 PMC4914639

[27] Li X, Fu Y, Shen M, Huang D, Du X, Hu Q, Zhou Y, Wang D, Yu Y 2018 Dissemination of *bla*_NDM-5_ gene via an IncX3-type plasmid among non-clonal *Escherichia coli* in China. Antimicrob Resist Infect Control 7:59. doi:10.1186/s13756-018-0349-6. 29713466 PMC5918551

[28] Brinkac LM, White R, D’Souza R, Nguyen K, Obaro SK, Fouts DE 2019 Emergence of New Delhi metallo-β-lactamase (NDM-5) in *Klebsiella quasipneumoniae* from neonates in a Nigerian hospital. mSphere 4:e00685-18. doi:10.1128/mSphere.00685-18. 30867330 PMC6416368

[29] Liu PP, Liu Y, Wang LH, Wei DD, Wan LG 2016 Draft genome sequence of an NDM-5-producing *Klebsiella pneumoniae* sequence type 14 strain of serotype K2. Genome Announc 4:e01610-15. doi:10.1128/genomeA.01610-15. 26988061 PMC4796140

[30] Potron A, Nordmann P, Lafeuille E, Al Maskari Z, Al Rashdi F, Poirel L 2011 Characterization of OXA-181, a carbapenem-hydrolyzing class D beta-lactamase from *Klebsiella pneumoniae*. Antimicrob Agents Chemother 55:4896–4899. doi:10.1128/AAC.00481-11. 21768505 PMC3186949

[31] Potron A, Rondinaud E, Poirel L, Belmonte O, Boyer S, Camiade S, Nordmann P 2013 Genetic and biochemical characterisation of OXA-232, a carbapenem-hydrolysing class D beta-lactamase from Enterobacteriaceae. Int J Antimicrob Agents 41:325–329. doi:10.1016/j.ijantimicag.2012.11.007. 23305656

[32] Pitart C, Solé M, Roca I, Román A, Moreno A, Vila J, Marco F 2015 Molecular characterization of *bla*_NDM-5_ carried on an IncFII plasmid in an *Escherichia coli* isolate from a nontraveler patient in Spain. Antimicrob Agents Chemother 59:659–662. doi:10.1128/AAC.04040-14. 25313215 PMC4291412

[33] Marando R, Seni J, Mirambo MM, Falgenhauer L, Moremi N, Mushi MF, Kayange N, Manyama F, Imirzalioglu C, Chakraborty T, Mshana SE 2018 Predictors of the extended-spectrum-beta lactamases producing *Enterobacteriaceae* neonatal sepsis at a tertiary hospital, Tanzania. Int J Med Microbiol 308:803–811. doi:10.1016/j.ijmm.2018.06.012. 29980372 PMC6171784

[34] Labi AK, Bjerrum S, Enweronu-Laryea CC, Ayibor PK, Nielsen KL, Marvig RL, Newman MJ, Andersen LP, Kurtzhals JAL 2020 High carriage rates of multidrug-resistant gram-negative bacteria in neonatal intensive care units from Ghana. Open Forum Infect Dis 7:ofaa109. doi:10.1093/ofid/ofaa109. 32373647 PMC7192099

[35] Wisgrill L, Lepuschitz S, Blaschitz M, Rittenschober-Böhm J, Diab-El Schahawi M, Schubert S, Indra A, Berger A 2019 Outbreak of Yersiniabactin-producing *Klebsiella pneumoniae* in a neonatal intensive care unit. Pediatr Infect Dis J 38:638–642. doi:10.1097/INF.0000000000002258. 30489463

[36] Wiegand I, Hilpert K, Hancock REW 2008 Agar and broth dilution methods to determine the minimal inhibitory concentration (MIC) of antimicrobial substances. Nat Protoc 3:163–175. doi:10.1038/nprot.2007.521. 18274517

[37] Clinical and Laboratory Standards Institute. 2020 Performance standards for antimicrobial susceptibility testing: thirtieth informational supplement M100-S30. CLSI, Wayne, PA.

[38] The European Committee on Antimicrobial Susceptibility Testing. 2020 Breakpoint tables for interpretation of MICs and zone Version 10.0. https://www.eucast.org/fileadmin/src/media/PDFs/EUCAST_files/Breakpoint_tables/v_11.0_Breakpoint_Tables.pdf.

[39] Naha S, Sands K, Mukherjee S, Roy C, Rameez MJ, Saha B, Dutta S, Walsh TR, Basu S 2020 KPC-2-producing *Klebsiella pneumoniae* ST147 in a neonatal unit: clonal isolates with differences in colistin susceptibility attributed to AcrAB-TolC pump. Int J Antimicrob Agents 55:105903. doi:10.1016/j.ijantimicag.2020.105903. 31954832

[40] Mitra S, Mukherjee S, Naha S, Chattopadhyay P, Dutta S, Basu S 2019 Evaluation of co-transfer of plasmid-mediated fluoroquinolone resistance genes and *bla*_NDM_ gene in Enterobacteriaceae causing neonatal septicaemia. Antimicrob Resist Infect Control 8:46. doi:10.1186/s13756-019-0477-7. 30858970 PMC6391786

[41] Feil EJ, Li BC, Aanensen DM, Hanage WP, Spratt BG 2004 eBURST: inferring patterns of evolutionary descent among clusters of related bacterial genotypes from multilocus sequence typing data. J Bacteriol 186:1518–1530. doi:10.1128/jb.186.5.1518-1530.2004. 14973027 PMC344416

[42] Tenover FC, Arbeit RD, Goering RV, Mickelsen PA, Murray BE, Persing DH, Swaminathan B 1995 Interpreting chromosomal DNA restriction patterns produced by pulsed-field gel electrophoresis: criteria for bacterial strain typing. J Clin Microbiol 33:2233–2239. doi:10.1128/JCM.33.9.2233-2239.1995. 7494007 PMC228385

[43] Kado CI, Liu ST 1981 Rapid procedure for detection and isolation of large and small plasmids. J Bacteriol 145:1365–1373. doi:10.1128/JB.145.3.1365-1373.1981. 7009583 PMC217141

[44] Carattoli A, Bertini A, Villa L, Falbo V, Hopkins KL, Threlfall EJ 2005 Identification of plasmids by PCR-based replicon typing. J Microbiol Methods 63:219–228. doi:10.1016/j.mimet.2005.03.018. 15935499

[45] Shibata N, Doi Y, Yamane K, Yagi T, Kurokawa H, Shibayama K, Kato H, Kai K, Arakawa Y 2003 PCR typing of genetic determinants for metallo-beta-lactamases and integrases carried by gram-negative bacteria isolated in Japan, with focus on the class 3 integron. J Clin Microbiol 41:5407–5413. doi:10.1128/jcm.41.12.5407-5413.2003. 14662918 PMC309014

[46] Novais A, Cantón R, Valverde A, Machado E, Galán JC, Peixe L, Carattoli A, Baquero F, Coque TM 2006 Dissemination and persistence of *bla*_CTX-M-9_ are linked to class 1 integrons containing CR1 associated with defective transposon derivatives from Tn*402* located in early antibiotic resistance plasmids of IncHI2, IncP1-alpha, and IncFI groups. Antimicrob Agents Chemother 50:2741–2750. doi:10.1128/AAC.00274-06. 16870767 PMC1538643

[47] Croucher NJ, Page AJ, Connor TR, Delaney AJ, Keane JA, Bentley SD, Parkhill J, Harris SR 2015 Rapid phylogenetic analysis of large samples of recombinant bacterial whole genome sequences using Gubbins. Nucleic Acids Res 43:e15. doi:10.1093/nar/gku1196. 25414349 PMC4330336

[48] Stamatakis A, Aberer AJ, Goll C, Smith SA, Berger SA, Izquierdo-Carrasco F 2012 RAxML-Light: a tool for computing terabyte phylogenies. Bioinformatics 28:2064–2066. doi:10.1093/bioinformatics/bts309. 22628519 PMC3400957

